# Oral health indicators and dental care utilization patterns among adults in a context of social vulnerability (Polígono Sur, Seville)

**DOI:** 10.3389/froh.2026.1840742

**Published:** 2026-06-03

**Authors:** Camila Fajardo, Diego Rodríguez Menacho, Alejandro Moreno Barrera, Ignacio Barbero Navarro, Antonio Castaño-Séiquer, David Ribas-Pérez

**Affiliations:** Facultad de Odontología, Universidad de Sevilla, Sevilla, Spain

**Keywords:** DMFT index, poligono sur, social exclusion, tooth brushing, vulnerable population

## Abstract

**Introduction:**

Access to quality dental services continues to be a challenge for individuals living in contexts of social exclusion. In the Polígono Sur area of Seville, the high socioeconomic vulnerability of the population highlights the need to identify barriers to access and to provide evidence for the development of more inclusive public policies.

**Objective:**

To describe the oral health status of a group of adults enrolled at the CEPer Polígono Sur, considering sociodemographic, clinical, and access-to-care aspects.

**Materials and methods:**

A descriptive cross-sectional study was conducted among 120 adults enrolled at the Centro de Educación Permanente (CEPer) Polígono Sur. Data were collected using an oral health survey based on the model proposed by the World Health Organization (WHO).

**Results:**

Ninety-four percent of the sample reported brushing their teeth at least once per day. Thirty percent had not visited a dentist recently, and 10% had never visited one, mainly individuals from Africa. Fifty-three percent experienced oral discomfort with varying intensity and frequency during the past year, of whom 17% reported it as “very frequent.” The overall DMFT index was 7.65. Forty-eight percent of the sample required some type of prosthetic treatment, particularly those aged 45–64 years.

**Conclusions:**

The demand for preventive education in this neighborhood is high. The overall experience of dental caries among adults is relatively low, with a high proportion of untreated dental caries (D component of DMFT) in younger individuals and extractions among older adults. Nearly half of the population experiences unmet prosthetic needs and lack of oral rehabilitation.

## 1 Introduction

Oral health is an essential component of overall health and individual well-being. In 2016, the World Dental Federation defined it as multifaceted, encompassing the ability to speak, smile, smell, taste, touch, chew, swallow, and convey a range of emotions through facial expressions with confidence and without pain or discomfort in the craniofacial complex. This definition extends far beyond the mere absence of oral disease (1).

Furthermore, the World Health Organization (WHO) warns that oral diseases represent a significant global public health challenge, with a particularly high incidence among vulnerable and marginalized groups who frequently encounter multiple barriers to accessing basic dental services. The WHO states that “there is a very clear and consistent relationship between socioeconomic status (income, occupation, and level of education) and the prevalence and severity of oral diseases […] across all population groups and in countries of all income levels” (2).

Oral health cannot be understood solely from a clinical or individual perspective. A growing body of research suggests that an individual's oral health status is heavily conditioned by the so-called social determinants of health: “the social circumstances in which people are born, grow, live, work, and age, which are essential to explaining the distribution of the risk of disease, disability, and/or death” 3, 4).

In line with the previous mentioned, it can be inferred that accessing quality dental services remains a challenge for many, especially in contexts of social exclusion. Factors such as limited economic resources, low health literacy, and cultural barriers can restrict access to adequate care (5). Consequently, it is imperative to study the oral health status of disadvantaged groups to detect disparities and propose inclusive strategies that promote equitable preventive care.

Socioeconomic status is one of the most influential factors. Various studies demonstrate that individuals with lower incomes exhibit poorer oral health and more limited access to both preventive and curative treatments (6). According to Montero (7), economic exclusion in Spain has a significant impact on obtaining oral health care, as public system coverage for adults is virtually non-existent beyond extractions, diagnostics, biopsies, and minor wisdom tooth surgeries. Remaining treatments must be privately funded by the citizen, leading to profound disparities based on the population's socioeconomic level.

Additionally, other determinants exert influence, such as educational attainment—which affects knowledge of oral hygiene habits and the perceived importance of regular check-ups—the social and family environment, and even ethnic and cultural background, which may hinder access to information and care due to linguistic or administrative reasons (8).

In contexts marked by exclusion, such as the Polígono Sur in Seville, these determinants take on particular relevance. “This neighborhood is perhaps the primary exponent of a socially and economically degraded area in the city of Seville, classified as a Zone in Need of Social Transformation (ZNTS), characterized by economic instability, unemployment, multi-ethnicity, and high levels of drug addiction and crime” (9).

According to the National Institute of Statistics (INE) in its Household Income Distribution Atlas (10), the Polígono Sur of Seville occupies the lowest income bracket (€15,640.4—€24,422.1 per year), ranking as one of the most impoverished neighborhoods in Spain. The selection of Polígono Sur as the scope of study addresses the need to approach oral health from a social perspective in contexts where unfavorable structural conditions directly impact health, particularly in aspects as frequently overlooked as dental care. This neighborhood represents a paradigmatic scenario for analyzing the relationship between social exclusion, oral health, and access to dental services (11).

Given the high socioeconomic vulnerability of this population, it is a priority to conduct studies that accurately describe the state of oral health in this environment, discuss potential obstacles to dental care utilization in this context, and provide evidence for the design of more inclusive public policies (12). This research aims to provide a rigorous description of the oral health status of adults at the CEPer Polígono Sur, reinforcing the need to consider oral health as a fundamental right within the healthcare system.

The objective of this study was to describe key oral health indicators (caries experience and prosthetic needs) and self-reported patterns of dental service utilization in a group of adults at the CEPer Polígono Sur, exploring how these factors relate to their sociodemographic profile.

## 2 Materials and methods

### 2.1 Study design and settings

The present research consists of a descriptive, cross-sectional study aimed at describing the oral health status of a group of adult students.

The study was conducted at the Centro de Educación Permanente (CEPer) Polígono Sur, located in the Polígono Sur neighborhood in the south-southeast district of Seville, Spain. This neighborhood faces significant social, economic, and healthcare challenges, being classified as one of the most disadvantaged areas in Spain (10). Data collection was carried out between January and June 2024.

### 2.2 Population and sample

The target population comprised adult students aged 16 years and older. A non-probabilistic convenience sampling method was employed, including all participants attending the CEPer during the study period who met the inclusion criteria and provided voluntary informed consent. The final sample consisted of 120 students. There were inclusion and excluson criteria for the sample (Table 1).

**Table 1:** Inclusion and exclusion criteria for the sample.

Inclusion Criteria	Exclusion Criteria
Individuals aged 16 years or more enrolled at CEPer Polígono Sur.	Participants who did not consent to the clinical evaluation.
Provision of voluntary informed consent (signed form).	Inability to undergo oral examination (clinical or personal reasons).
Willingness to pa rticipate in the full clinical assessment.	Incomplete surveys or non-recordable clinical data.

A convenience sampling method was employed due to the specific challenges associated with conducting research in highly marginalized and “hard-to-reach” contexts. Polígono Sur is characterized by significant social fragmentation and trust barriers toward external institutions; therefore, the Adult Education Center (CEPer) provided a safe, stable, and ethically sound environment to approach participants. We think that this approach prioritizes internal validity and feasibility over broad population representativeness.

During the study period, approximately 160 students were invited to participate. Of these, 132 expressed initial interest, and 120 (75% of the total invited) finally met the inclusion criteria and completed both the survey and the clinical examination. The remaining 12 were excluded due to incomplete clinical records or withdrawal of consent during the process. No imputation methods were used for missing data.

### 2.3 Instruments and data collection

Data were obtained using an oral health survey based on the model proposed by the WHO. For the diagnosis of dental caries, the DMFT index (Decayed, Missing, and Filled Teeth) was used. This internationally accepted index assesses caries experience and presence, ensuring the standardization of results and allowing for the comparison of data with other studies. The same book includes a questionnaire on access to dental care, as well as perceptions of pain and the use of dentures, which was also used (13). Prosthetic status was assessed following WHO criteria. A prosthesis was defined as “functional” if it provided adequate retention and stability during examination and was in a good state of repair. It was categorized as “dysfunctional” if it showed fractures, extreme wear, or lack of stability that compromised its clinical purpose.

The clinical examination was performed by a single calibrated clinical examiner with the patient seated in a high-backed chair, head extended, and using frontal lighting with the examiner at a distance of 25 cm. Data were recorded by another member of the dental team to ensure accuracy and minimize bias. Before data collection, the examiner underwent specific training to standardize the assessment of the DMFT index and prosthetic needs. To ensure the reliability of the data, an intra-examiner calibration was performed by re-evaluating 12 participants (10% of the sample) after a two-week interval, achieving a Cohen's Kappa coefficient of 0.90, which reflects high diagnostic consistency.

The clinical examination focused exclusively on the assessment of dental caries (DMFT index) and prosthetic status. Periodontal health and soft tissue lesions were considered beyond the initial scope of this study. This decision was made to prioritize the identification of the most acute rehabilitative needs and to ensure the feasibility of the examinations within the logistical constraints of the educational center. By narrowing the clinical focus, the study was able to achieve a higher participation rate and ensure standardized data collection for the primary outcomes of interest.

### 2.4 Ethical considerations

The study adhered to the fundamental ethical principles of health research: autonomy, confidentiality, beneficence, and non-maleficence. Informed consent was obtained from all participants, guaranteeing their right to voluntary participation. The collected information was used exclusively for academic purposes, ensuring the anonymity of the subjects. Ethical guidelines of the Declaration of Helsinki were followed. The study was approved by the Ethics Committee of the Social Dentistry Foundation (FOS) under registration number 03/2024.

### 2.5 Data analysis

Data were recorded and organized in a spreadsheet using Microsoft Excel 365. For processing and analysis, the statistical software SPSS (version 27.0) was employed. Both descriptive and inferential statistical techniques were applied to explore potential relationships between variables of interest (age, number of decayed, filled, and missing teeth) and associated factors, such as the frequency of dental pain and dental visits.

Initially, a descriptive analysis of quantitative and categorical variables was performed to characterize the sample. Subsequently, Pearson correlation tests were applied to study lineal relationships between numerical variables. Data normality was verified using the Shapiro–Wilk test, and homogeneity of variances was assessed via Levene's test.

Furthermore, a one-way analysis of variance (ANOVA) was used to identify significant differences in the means of oral variables across categories if normality was verified otherwise, the Kruskal–Wallis *H*-test was applied. Statistical significance was set at *p* < 0.05 with a 95% confidence level, with *p* < 0.01 considered highly significant.

## 3 Results

### 3.1 Sociodemographic profile of the population

**Figure 1:**
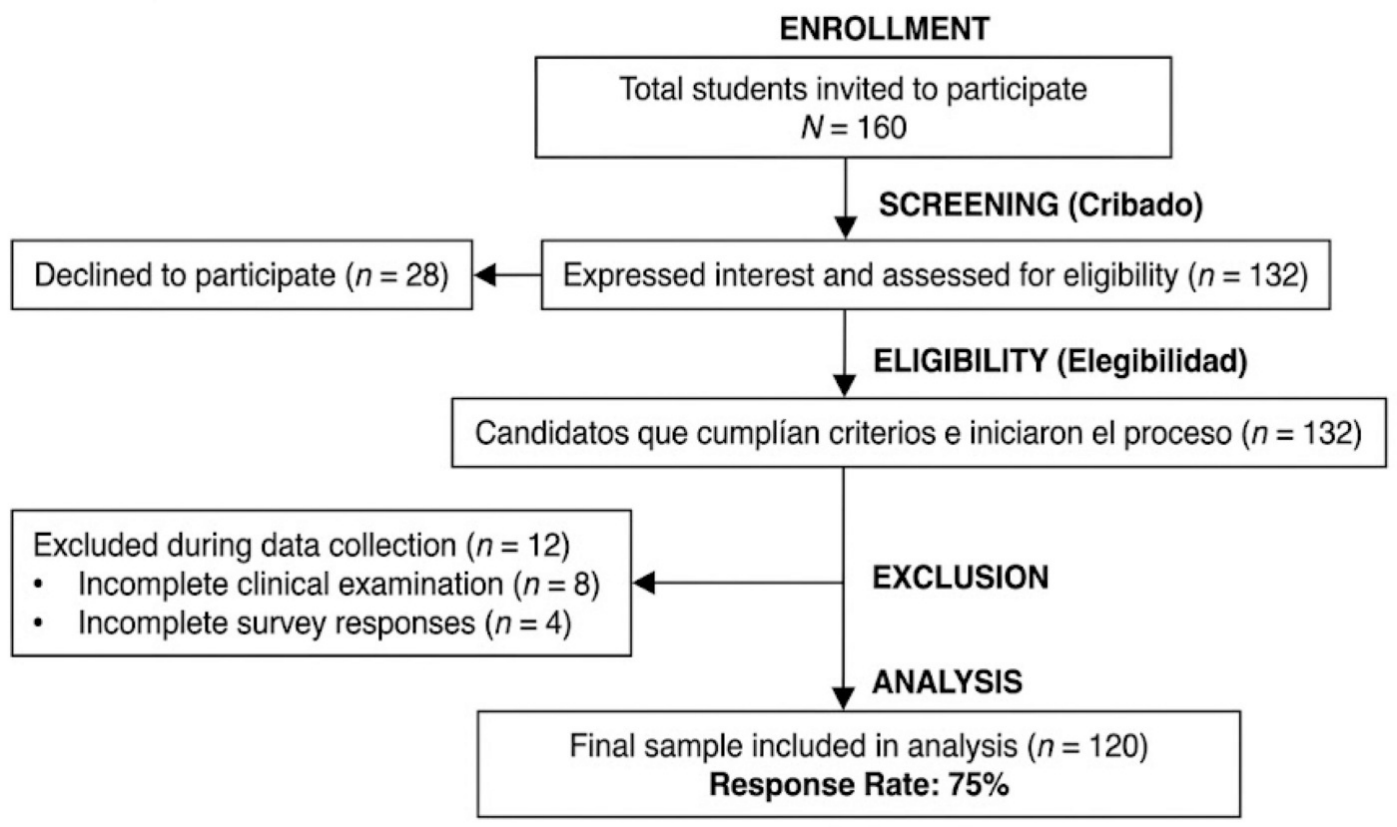
Participant flow diagram. STROBE guidelines.

The participant recruitment and flow are summarized in Figure 1. A total of 160 students from the Adult Education Center (CEPer) were initially invited to participate in the study. Of these, 132 individuals expressed interest and were screened for eligibility. Twelve individuals were excluded: 8 did not complete the clinical examination due to time constraints, and 4 provided incomplete surveys. The final analytical sample consisted of 120 participants, resulting in a 75% response rate (120/160).

The final study population consisted of 120 adults enrolled at the CEPer Polígono Sur in Seville. The age distribution reveals a higher participation of individuals in the active working age group (25–44 years, *n* = 48), followed by young adults (17–24 years, *n* = 36). Middle-aged (45–64 years, *n* = 24) and older adults (65+ years, *n* = 12) represented smaller proportions of the sample. Regarding gender, there was a female predominance (65% females vs. 35% males). The participants exhibited significant multicultural diversity: 65% of Spanish origin, 22% African origin, 8% Latin American origin, and 5% from the European Union (excluding Spain). Overall, 35% of the sample was of non-Spanish origin.

### 3.2 Oral hygiene habits

As shown in Table 2, 94% of the participants reported brushing their teeth at least once a day. Notably, 100% of male participants brush at least once daily, whereas 3% of females reported never brushing. Participants from the European Union (EU) and Latin America reported the highest frequency of oral hygiene.

**Table 2:** Frequency of oral hygiene habits by subgroup.

Subgroup	*n*	Never/Rarely *n* (%)	≥1/day *n* (%)	>1/day *n* (%)	Total Daily *n* (%)
Total	120	8 (7%)	44 (37%)	68 (56%)	112 (93%)
Gender					
Male	42	0 (0%)	18 (43%)	24 (57%)	42 (100%)
Female	78	8 (10%)	26 (33%)	44 (56%)	70 (89%)
Age Group					
17–24 years	36	2 (6%)	14 (39%)	20 (55%)	34 (94%)
25–44 years	48	6 (12%)	16 (33%)	26 (54%)	42 (88%)
45–64 years	24	0 (0%)	8 (33%)	16 (67%)	24 (100%)
65+ years	12	0 (0%)	6 (50%)	6 (50%)	12 (100%)
Origin					
Spain	78	6 (8%)	32 (41%)	40 (51%)	72 (92%)
EU	6	0 (0%)	2 (33%)	4 (67%)	6 (100%)
Latin America	10	0 (0%)	0 (0%)	10 (100%)	10 (100%)
Africa	26	2 (8%)	10 (38%)	14 (54%)	24 (92%)

### 3.3 Dental service utilization

30% of participants had not visited a dentist in the last 12 months. This percentage was higher among the 25–44 age group (38%), individuals of African (38%) and Latin American origin (40%), and males (33%) (Table 3).

**Table 3:** Frequency of dental services utilization by subgroup.

Subgroup	*n*	No visit/Never *n* (%)	1 visit *n* (%)	2 visits *n* (%)	3 + visits *n* (%)	Total Utilized *n* (%)
Total	120	48 (40%)	30 (25%)	16 (13%)	26 (22%)	72 (60%)
Gender						
Male	42	20 (47%)	6 (14%)	8 (19%)	8 (19%)	22 (52%)
Female	78	28 (36%)	24 (31%)	8 (10%)	18 (23%)	50 (64%)
Age Group						
17–24 years	36	16 (44%)	10 (28%)	4 (11%)	6 (17%)	20 (56%)
25–44 years	48	22 (46%)	10 (21%)	10 (21%)	6 (13%)	26 (54%)
45–64 years	24	8 (33%)	8 (33%)	0 (0%)	8 (33%)	16 (67%)
65 + years	12	2 (17%)	2 (17%)	2 (17%)	6 (50%)	10 (83%)
Origin						
Spain	78	26 (34%)	22 (28%)	10 (13%)	20 (26%)	52 (67%)
EU	6	2 (33%)	2 (33%)	0 (0%)	2 (33%)	4 (67%)
Latin America	10	4 (40%)	0 (0%)	2 (20%)	4 (40%)	6 (60%)
Africa	26	16 (61%)	6 (23%)	4 (15%)	0 (0%)	10 (39%)

### 3.4 Prevalence of dental pain and discomfort

75% of the sample reported experiencing some degree of oral pain or discomfort during the last 12 months, with 37% reporting frequent or very frequent pain. Females reported higher levels of “very frequent” pain (23% vs. 5% in males) (Table 4).

**Table 4:** Frequency of dental pain and discomfort by subgroup.

Subgroup	n	Never/Rarely *n* (%)	Sometimes *n* (%)	Often/Very Freq. *n* (%)
Total	120	56 (47%)	20 (17%)	44 (37%)
Gender				
Male	42	24 (58%)	6 (14%)	12 (28%)
Female	78	32 (41%)	14 (18%)	32 (41%)
Age Group				
17–24 years	36	18 (50%)	6 (17%)	12 (33%)
25–44 years	48	20 (42%)	8 (17%)	20 (42%)
45–64 years	24	6 (25%)	6 (25%)	12 (50%)
65+ years	12	12 (100%)	0 (0%)	0 (0%)

### 3.5 DMFT index (caries, missing, and filled teeth)

**Figure 2:**
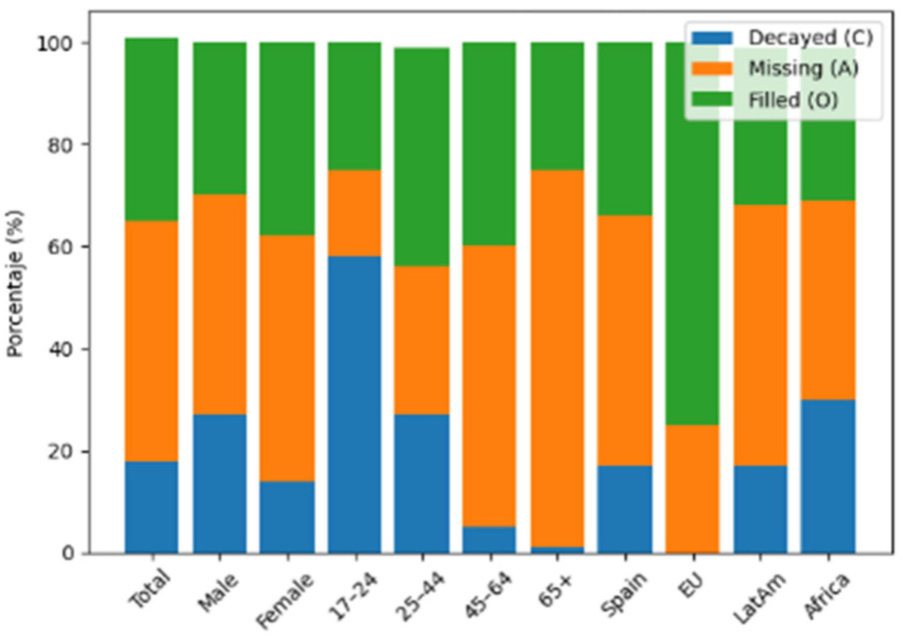
DMFT Index and distribution of components by subgroup.

The mean DMFT index for the total population was 7.65. The composition of the index was as follows: 18% decayed teeth (C), 47% missing teeth (M), and 36% filled teeth (F). According to WHO classifications for adults, the sample shows a predominantly low-to-moderate level of cumulative oral damage (Table 5, Figure 2).

**Table 5:** DMFT Index and distribution of components by subgroup.

Subgroup	*n*	Mean DMFT	Decayed (C)	Missing (A)	Filled (O)
Total	120	7.65	18%	47%	36%
Male	42	6.70	27%	43%	30%
Female	78	8.10	14%	48%	38%
17–24 yrs	36	3.30	58%	17%	25%
25–44 yrs	48	6.70	27%	29%	43%
45–64 yrs	24	13.10	5%	55%	40%
65 + yrs	12	17.50	1%	74%	25%
Spain	78	9.10	17%	49%	34%
EU	6	8.00	0%	25%	75%
Latin America	10	7.00	17%	51%	31%
Africa	26	3.50	30%	39%	30%

### 3.6 Prosthetic treatment needs

Table 6 highlights that 48% of the sample requires prosthetic treatment but has never received it. Only 12% of the participants were found to be functionally rehabilitated.

**Table 6:** Prosthetic use and needs.

Subgroup	*n*	No need *n* (%)	Unmet Need *n* (%)	Functional *n* (%)	Dysfunctional *n* (%)
Total	120	46 (38%)	58 (48%)	14 (12%)	2 (2%)
Male	42	18 (43%)	16 (38%)	8 (19%)	0 (0%)
Female	78	28 (36%)	42 (54%)	6 (8%)	2 (3%)
Age Group					
17–24 years	36	26 (72%)	10 (28%)	0 (0%)	0 (0%)
25–44 years	48	20 (42%)	24 (50%)	4 (8%)	0 (0%)
45–64 years	24	0 (0%)	20 (83%)	4 (17%)	0 (0%)
65 + years	12	0 (0%)	4 (33%)	6 (50%)	2 (17%)

### 3.7 Statistical analysis (correlations)

Statistical analysis using one-way ANOVA was performed to examine associations between variables. A significant association was observed between pain perception and the number of decayed teeth (*p* = 0.034), suggesting that symptomatic discomfort is a primary indicator of untreated caries. However, no statistically significant relationship was found between the frequency of dental visits and the number of decayed teeth (*p* = 0.244).

When we examine the relationship between age and the different categories of the DMFT, a strong correlation stands out between age and the performance of invasive procedures (tooth extractions) (*r* = 0.781, *p* < 0.001), whereas this correlation is not as strong for fillings (r = 0.506, *p* < 0.001). (Table 7).

**Table 7:** Bivariate analysis of clinical indicators and self-reported health factors.

Variable Relationship	F-value	Significance (p)	Interpretation	Effect size (eta^2)	95% CI (Mean D
Pain Perception vs. Decayed Teeth	2.808	0.034*	Statistically Significant	0.08 (Medium)	[1,42–2,98]
Dental Visits vs. Decayed Teeth	1.405	0.244	Not Significant	0.03 (Small)	[1.80–2.60]
Dental Visits vs. Filled Teeth	1.784	0.145	Not Significant	0.04 (Small)	[1.10–1.95]

* Statistical significance for *p*<0.05.

## 4 Discussion

While this study does not perform a comparative analysis between different socioeconomic gradients to formally quantify health inequalities, the descriptive data regarding high untreated caries and low service utilization are highly suggestive of the structural challenges faced by this population. These findings should be considered contextual hypotheses that warrant further investigation through comparative analytical designs.

The use of convenience sampling introduces a clear risk of selection bias that must be acknowledged. The participants, being students at an adult education center, may possess a higher degree of social integration, cognitive motivation, or health awareness compared to the most marginalized residents of Polígono Sur who do not engage with such institutions. Consequently, our findings may underestimate the severity of oral health disparities in the broader neighborhood. These results should therefore be interpreted as a descriptive baseline for this specific sub-community rather than a strictly representative profile of the entire district's population.

### 4.1 Sociodemographic profile

The study population (*n* = 120) at the Centro de Educación Permanente (CEPer) Polígono Sur displays a heterogeneous age distribution, providing a comprehensive overview of oral health across distinct adulthood stages. The largest cohort (40%) comprises individuals in the active working-age group (25–44 years), followed by young adults (17–24 years) at 30%. Middle-aged (45–64 years) and older adults (65+) account for 20% and 10% of the sample, respectively.

This demographic diversity allows for an in-depth analysis of age-dependent oral health requirements within an educational setting (11). Regarding gender, the female predominance (65%) may reflect varying levels of health literacy or differential engagement in self-care and lifelong learning initiatives as int´s been reported in similar studies (12).

The multicultural composition of the Polígono Sur neighborhood is reflected in the sample, where 35% of participants are of non-Spanish origin, with the largest subgroup being of African descent (22%), followed by Latin American (8%) and European Union nationals (5%). This cultural heterogeneity aligns with the “South-South migration” framework described by Murillo-Pedrozo and Agudelo-Suárez (14), which posits that migratory flows between regions with fragile social structures exacerbate health disparites. In this context, migrant populations—particularly those of African origin—face cumulative disadvantages, including labor precariousness, restricted healthcare access, linguistic barriers, and a lack of culturally adapted public health policies (15).

### 4.2 Health determinants

#### 4.2.1 Oral hygiene habits

Overall, 94% of participants reported brushing their teeth at least once daily. However, 6% exhibit high-risk hygiene patterns, including those who brush infrequently or never. Notably, while 100% of male participants report daily brushing, the DMFT index suggests they experience higher caries prevalence than females, potentially indicating less effective brushing techniques.

Adherence to daily hygiene habits improves with age; notably, the 45+ group demonstrates 100% daily brushing compliance. The observed patterns of oral hygiene in Polígono Sur are consistent with studies on populations facing social exclusion, where structural factors—such as limited access to dental hygiene products or the prioritization of basic survival needs over preventive health—predominate (9, 11, 16).

#### 4.2.2 Utilization of dental services

While access to healthcare is a multidimensional construct involving availability and affordability, this study focuses specifically on utilization patterns and perceived barriers such as pain. Although the clinical assessment was centered on the DMFT index and prosthetic needs—common indicators in WHO-based surveys—we recognize that a comprehensive oral health status would require further periodontal and soft tissue analysis. Nonetheless, the indicators presented here provide a critical baseline for a population that is traditionally invisible in official health statistics.

Access to dental care remains suboptimal, with 30% of the sample reporting no visits in the past 12 months and 10% having never accessed dental services. This indicates a healthcare utilization pattern driven by symptomatic necessity rather than preventive oversight.

Younger participants (17–24 years) exhibit the highest rate of never having visited a dentist (22%), whereas older adults show higher attendance. Among African-origin participants, 61% reported no utilization of services in the last year or ever, marking the lowest utilization rates among all subgroups. These findings mirror national trends in disadvantaged social classes (17, 18), reinforcing the conclusion that social exclusion remains a primary driver of oral health disparity.

Similarly, a U.S. study reaches conclusions similar to ours, as people in the U.S. who are low-income, uninsured, or part of marginalized groups (including racial/ethnic minorities, immigrants, and rural populations) are more likely to experience poor oral health due to limited access to quality care. This makes oral health a visible indicator of broader social inequality. Public health experts increasingly recognize that oral diseases (like cavities and gum disease) are closely connected to overall health conditions such as obesity and diabetes. These conditions share common risk factors, including high sugar intake or tobacco use (19).

### 4.3 Clinical status and associated needs

#### 4.3.1 Perception of pain and oral discomfort

53% of participants reported experiencing oral pain or discomfort within the last 12 months, with 17% classifying this as “very frequent.” This high symptom prevalence is likely associated with untreated conditions, including caries and periodontal disease. Women reported higher frequencies of “very frequent” pain (23%) compared to men (5%). Furthermore, individuals of African and Latin American origin reported significantly higher pain frequencies, suggesting that vulnerability and systemic exclusion manifest as persistent clinical suffering.

Statistical analysis confirmed a clear progression: higher frequency of pain is positively correlated with a higher number of decayed teeth. This fact can be named as The Symptom-Disease Link: A significant positive association (*p* = 0.034) was found between the frequency of reported pain and the number of carious lesions. This indicates that self-reported discomfort is a reliable clinical indicator of untreated caries in this population (20, 21). The association between pain perception and untreated caries (decayed teeth) not only reached statistical significance (*p* = 0.034) but also demonstrated a medium effect size (η^2^ = 0.08). This reinforces the hypothesis that in vulnerable populations, dental service utilization is predominantly reactive, with clinical status being moderately tied to acute symptomatic episodes rather than preventive routines.

#### 4.3.2 DMFT Index

The distribution of the DMFT index components (47% Missing and 36% Filled) provides a nuanced picture of dental care utilization. While the high proportion of missing teeth suggests a history of radical treatments, the presence of filled teeth indicates that a significant portion of the sample has had prior access to dental services. This suggests that the issue may not be an absolute lack of access, but rather a pattern of care that is potentially reactive or delayed, occurring when conditions are advanced. These findings are consistent with what we observed in our statistical analysis, where pain—rather than prevention—appears to be the primary driver for seeking care.

The mean DMFT index of 7.65 indicates a low-to-moderate level of cumulative oral damage. However, the high prevalence of missing teeth (47%) highlights a reliance on radical treatments (extractions) over preventive or restorative interventions. Age-related increases in DMFT were significant, with the 65+ cohort reaching a “severe” index of 17.5. In the younger cohort (17–24 years), the D component represents 58% of the total DMFT index. While the DMFT reflects cumulative experience, this high percentage of the “D” component specifically indicates a significant burden of untreated cavitated lesions at the time of the study, rather than a history of restorative care.

Statistical analysis revealed a strong positive correlation between age and the number of missing teeth (*r* = 0.781, *p* < 0.01), as well as between age and the number of filled teeth (*r* = 0.506, *p* < 0.01). These results suggest that while restorative care is provided, it is often insufficient to prevent progressive tooth loss. It is the so called Service Utilization Paradox: the current pattern of dental service utilization in the Polígono Sur may not be effectively translating into a reduced burden of disease or increased restorative coverage, likely due to the reactive nature of the care received (i.e., extractions vs. comprehensive restorative or preventive treatment) (22).

The strong positive correlation between age and missing teeth (*r* = 0.781) and filled teeth (*r* = 0.506) must be interpreted with caution. Rather than indicating superior access to care in older cohorts, these figures likely reflect the cumulative burden of oral disease and the lifetime exposure to dental interventions. In high-vulnerability contexts, tooth loss (missing teeth) often represents the final stage of a long history of untreated disease, whereas the presence of fillings reflects sporadic past treatments rather than a consistent pattern of preventive care.

#### 4.3.3 Prosthetic rehabilitation

Nearly half of the sample (48%) requires prosthetic treatment but has never accessed it, reflecting a severe disparity in oral healthcare. This finding is consistent with recent data (7) identifying unmet prosthetic needs in 40%–50% of socially excluded populations. The need for prosthetic rehabilitation increases with age, yet access remains minimal across all studied strata, with the exception of participants from the EU.

The absence of prosthetic rehabilitation not only compromises masticatory function and speech but also precipitates secondary issues such as malnutrition and social isolation, particularly among older adults and those in irregular employment. These findings confirm that within Polígono Sur, health exclusion is deeply intertwined with broader social and educational disparities (18, 19, 22, 23).

While the current study identifies significant correlations between age, pain perception, and clinical status, a predictive risk analysis through logistic regression was not performed. Given the exploratory nature of this research and the specific sample size (*n* = 120), the authors prioritized a robust descriptive and correlational approach to establish a baseline for this underserved population. Over-modeling with multivariate regressions in this context could lead to over-interpretation of specific risk factors. Future studies with larger, stratified samples will be essential to calculate Odds Ratios (OR) that can precisely quantify the weight of social determinants on oral health in Polígono Sur.

### 4.4 Study limitations

This research has limitations that must be considered. First, the use of a non-probabilistic convenience sample from an educational center introduces a selection bias; participants may have higher levels of health literacy or social motivation than the general population of Polígono Sur. Therefore, these results should be interpreted as representative of this specific educational cohort rather than the neighborhood's entire population. Second, the lack of a formal sample size calculation limits the generalizability of the findings, although the sample (*n* = 120) is comparable to other exploratory studies in similarly vulnerable and hard-to-reach contexts.

Data regarding hygiene habits and dental service utilization rely on self-reported information, which is susceptible to social desirability bias and recall bias. This could lead to an overestimation of “positive” health behaviors, such as brushing frequency.

We also assume the small sample sizes in certain subgroups (e.g., participants from the EU or Latin America) result in unstable denominators. While these data are valuable for descriptive purposes, the percentages in these categories should be interpreted with caution as they are sensitive to individual variations.

The external validity (generalizability) of the study is limited. As a cross-sectional study focused on a specific sub-population within one neighborhood, the findings cannot be extrapolated to the entire population of Polígono Sur or to other vulnerable contexts. However, despite these limitations, the study provides a critical and rare clinical baseline for a traditionally “hard-to-reach” population, highlighting urgent unmet dental needs.

Our results show high levels of untreated disease and unmet prosthetic needs but these findings should be interpreted cautiously within the specific context of Polígono Sur. Although structural exclusion and cultural barriers are plausible factors that align with the neighborhood's socioeconomic profile, our study was not designed to establish a direct causal link between these mechanisms and the participants' oral health. Future qualitative research is needed to directly explore the lived experiences and specific barriers perceived by this population.

## 5 Conclusions

This study provides empirical evidence of the profound oral health disparities affecting the Polígono Sur community in Seville. The findings reveal a clear paradox related to the service use: while oral hygiene habits are self-reported as high, clinical indicators like the DMFT index and the 48% rate of unmet prosthetic needs highlight a reactive, rather than preventive, pattern of care.

Socioeconomic and cultural factors—particularly among the migrant population—act as significant barriers to access, leading to a high prevalence of untreated dental pain. To address these disparities, it is essential to move beyond basic extractions and implement inclusive public policies that provide preventive, restorative and prosthetic coverage, integrating these vulnerable groups into a more equitable healthcare framework.

## Data Availability

The raw data supporting the conclusions of this article will be made available by the authors, without undue reservation.
